# Impact of the COVID-19 lockdown on the adherence of stroke patients to direct oral anticoagulants: a secondary analysis from the MAAESTRO study

**DOI:** 10.1007/s00415-021-10631-5

**Published:** 2021-06-03

**Authors:** Fine Dietrich, Alexandros A. Polymeris, Melina Verbeek, Stefan T. Engelter, Kurt E. Hersberger, Sabine Schaedelin, Isabelle Arnet, Philippe A. Lyrer

**Affiliations:** 1 Pharmaceutical Care Research Group, Department of Pharmaceutical Sciences, University of Basel, Petersplatz 14, PO Box 2148, 4001 Basel, Switzerland; 2 Department of Neurology and Stroke Centre, University Hospital Basel and University of Basel, Basel, Switzerland; 3 Neurology and Neurorehabilitation, University Department of Geriatric Medicine Felix Platter, University of Basel, Basel, Switzerland; 4 Clinical Trial Unit, Department of Clinical Research, University Hospital Basel and University of Basel, Basel, Switzerland

**Keywords:** COVID-19, Ischaemic stroke, Medication adherence, Direct oral anticoagulation, Electronic monitoring

## Abstract

**Background:**

The negative impact of the COVID-19 outbreak on stroke care has been reported, but no data exist on the influence of the lockdown on medication adherence to antithrombotic treatment for stroke prevention. We present a comparison of electronic adherence data of stroke patients treated with direct oral anticoagulants (DOAC) prior to and during the COVID-19 lockdown in spring 2020 in Switzerland.

**Methods:**

This is a secondary analysis using data from the ongoing MAAESTRO study, in which stroke patients with atrial fibrillation electronically monitor their adherence to DOAC treatment. Eligible patients for this analysis had at least four weeks of adherence data prior to and during the COVID-19 lockdown. Three adherence metrics (*taking adherence, timing adherence, drug holidays*) were calculated and compared descriptively.

**Results:**

The analysis included eight patients (median age 81.5 years, IQR 74.8–84.5). Five patients had a pre-lockdown *taking adherence* over 90% (mean 96.8% ± 2.9), with no change during lockdown, high *timing adherence* in both periods and no *drug holidays*. The remaining three patients had pre-lockdown *taking* and *timing adherence *below 90%. Of those, two patients showed a moderate decline either in *taking* or *timing adherence* compared to pre-lockdown. One showed a substantial increase in *taking* and *timing adherence* during lockdown (both + 25.8%).

**Conclusion:**

Our data suggest that a major disruption of social life (i.e., the imposed COVID-19 lockdown) is unlikely to relevantly affect the medication intake behaviour of patients with high pre-established adherence, but might have an impact in patients with previously suboptimal adherence.

**Trial registration number:**

MAAESTRO: electronic Monitoring and improvement of Adherence to direct oral Anticoagulant treatment—a randomized crossover study of an Educational and reminder-based intervention in ischaemic STROke patients under polypharmacy, NCT03344146.

**Supplementary Information:**

The online version contains supplementary material available at 10.1007/s00415-021-10631-5.

## Introduction

The disease caused by the SARS-CoV-2 coronavirus (COVID-19) has severely disrupted social life and put an unprecedented strain on healthcare systems around the globe. Multiple reports demonstrated the negative impact of the COVID-19 outbreak on acute stroke care, including a decrease in stroke admissions [1–5] and a decline in the use of acute stroke imaging and the rate of reperfusion treatment [4–7]. Post-acute outpatient stroke care seems to have been similarly affected in the context of the general reduction in non-urgent care [8]. This included the interruption of care pathways focussing on secondary cardiovascular prevention [9, 10] with delayed or reduced stroke follow-ups [11, 12], and limitations in the access to rehabilitation services for stroke survivors [13].

While the adverse consequences of the pandemic on the acute and post-acute care of stroke patients are becoming apparent, data on the lockdown’s impact on the implementation of long-term secondary preventive therapies for stroke are scarce [14], and no data exist on its impact on the adherence to antithrombotic drugs.

Adherence to direct oral anticoagulants (DOACs) is of particular concern due to their short half-lives and the lack of coagulation monitoring [15]. Suboptimal adherence to DOACs, even seemingly minor deviations, have been associated with poor clinical outcomes [16–18]. Several factors might interfere with the adherence of stroke patients to DOACs in the context of the pandemic and the imposed lockdown measures [19], including the reduced contact to healthcare providers, interference with regular daily routine [20] and psychological factors [21].

Considering the lack of data on the impact of the COVID-19 pandemic on the adherence of stroke patients to DOACs, we present here for the first time detailed electronic adherence data of DOAC-treated stroke patients from the ongoing MAAESTRO study [22, 23], comparing various adherence metrics before and after the imposition of lockdown measures during the first COVID-19 outbreak in Switzerland in March 2020.

## Methods

### Study setting

The MAAESTRO study (electronic Monitoring and improvement of Adherence to direct oral Anticoagulant treatment—a randomized crossover study of an Educational and reminder-based intervention in ischaemic STROke patients under polypharmacy; NCT03344146) is an ongoing single-centre, randomized, crossover, open-label study that investigates an adherence-improving intervention in polymedicated, DOAC-treated patients with atrial fibrillation and a recent stroke who self-administer their medication in Basel, Switzerland. Participants self-monitor their adherence to DOACs during 12 months with a small electronic device [24]. The study includes an initial observational 6-month phase after discharge from the stroke hospitalization. The full study protocol [22] and first results of the observational study phase [23] have been published previously. The MAAESTRO study has been approved by the Ethics Committee of Northwest/Central Switzerland (EKNZ 2017-01552), including the use of study data for further investigations. All MAAESTRO participants gave written informed consent.

In Switzerland, a nationwide lockdown was imposed on March 16, 2020 that forced the population to stay at home, prohibited large gatherings and commercial activities, closed boarders and shut down most stores [25]. This lasted until June 19, 2020, when most of the measures were lifted [26]. Despite the lockdown, the MAAESTRO study continued its activities applying increased patient safety measures and rescheduling or switching clinical follow-up visits to telephone when applicable.

We performed a secondary analysis of MAAESTRO data from participants who (i) were enrolled in the observational study phase and were treated with DOACs at the time of the imposition of the lockdown and (ii) had adherence monitoring data over four weeks or more prior to and after the imposition of the lockdown.

### Adherence monitoring

MAAESTRO participants are asked to press a button on a small electronic device (Time4Med™, Adherence Innovations, Hong Kong, China) every time they ingest a DOAC [22, 23]. A beeping sound confirms the recording of the medication intake. We replace devices every month via post mailing to ensure data continuity. The monitoring period in the MAAESTRO study begins with the first electronic registration of a DOAC intake after patients’ discharge to their home from the acute or the rehabilitation hospital. For this secondary analysis, the last monitored day was either the end of the observational phase or the end of the COVID-19 lockdown in Switzerland (June 19, 2020) [26, 27], whichever occurred first. Using adherence data from this monitoring period we calculated various adherence metrics prior to (pre) and during the lockdown, as detailed below. As a sensitivity analysis, we repeated our calculations using the totality of the monitoring data from the observational phase (i.e., six months ± two weeks for each patient), independently of the end of the lockdown.

### Adherence estimates and questions on medication intake behaviour

We calculated three medication adherence metrics with the following formulas:

1. *Taking adherence* [28] [%] = (number of doses taken)/(number of prescribed doses) × 100;
2. *Timing adherence* [29] [%] = (number of doses taken within ± 25% of the median intake time [i.e., grace interval])/(number of prescribed doses) × 100;
3. *Drug holidays* [30] [number] = number of three or more consecutive days without medication intake.

Two questions were asked during the regular follow-up interviews that occurred at least six months after the lockdown was lifted at the end of the study, with focus on a possible impact of the lockdown on the participants’ medication intake behaviour:

- “Do you think that the COVID-19 lockdown changed anything about your medication intake behaviour?” (yes/no)
- “If yes, what has changed for you?” (free comment).

### Statistical analysis

We downloaded the adherence monitoring data from the devices with a tablet computer using near field communication, as described previously [23]. Data format was CSV files, which were saved on a secure server. Data cleaning was performed using Microsoft Excel® 2016 enriched by customized macros within a standardized template according to an internal standard operating procedure. The methods for complete data processing are described in detail elsewhere [23]. Adherence calculations were performed with Microsoft Excel® 2016 using a standardized template. Patients' adherence data prior to and during the COVID-19 lockdown were compared intra-individually using descriptive statistics. Adherence metrics and categorical values are reported as percentages. Continuous variables are reported as median with interquartile range (IQR) or mean with standard deviation (SD).

## Results

**Fig. 1:**
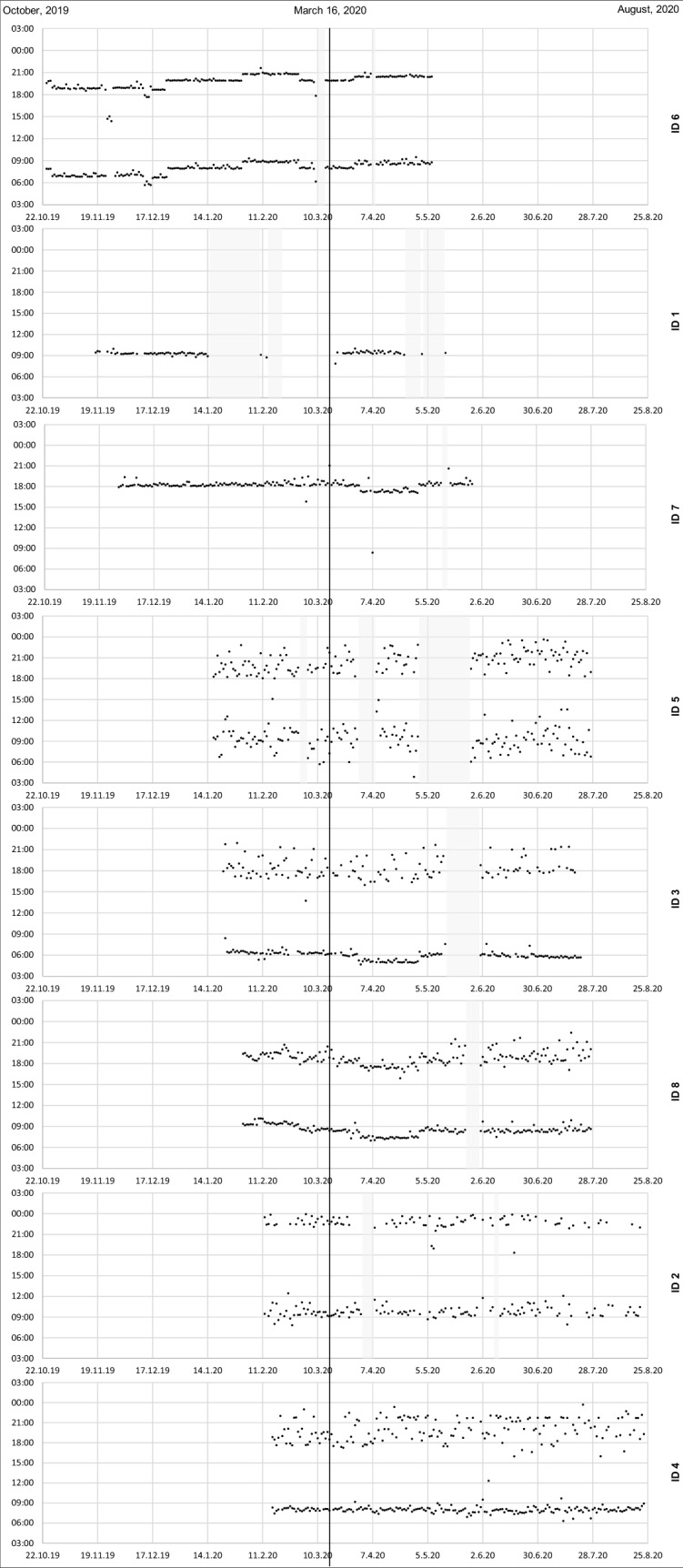
Individual dot charts of electronic data of eight patients in chronological order according to study entry, prior to and during the COVID-19 lockdown in Switzerland, that started on March 16, 2020 (black line). The patient ID is given on the right. Every dot represents the intake of a DOAC. The grey bars represent excluded days, either due to technical errors of the electronic devices or physician-initiated DOAC pauses

On March 16, 2020, at the day of the imposition of the lockdown, 12 patients actively participated in the observational phase of the MAAESTRO study. We excluded three patients who had less than four weeks of adherence data prior to the lockdown and one patient who had switched from DOAC to a vitamin K antagonist. Thus, the analysis was performed with eight patients whose electronic adherence data reached from October 24, 2019 up to August 24, 2020 (Fig. 1).

### Patient characteristics

The median age was 81.5 years (IQR 74.8–84.5) and five patients were male. The median NIHSS (National Institute of Health Stroke Scale) was 1.5 (IQR 0.8–2.3), median mRS (modified Rankin Scale) was 2.0 (IQR 1.8–2.0) and median MoCA (Montreal Cognitive Assessment) score was 26 (IQR 25–28). Six patients had a twice-daily DOAC regimen (apixaban: 4, dabigatran etexilate: 2) and two patients had a once-daily regimen (rivaroxaban: 1, edoxaban: 1; Table 1). Five patients used a pillbox as medication management strategy, and two of them had also installed an alarm on their mobile phones as intake reminder. No patient changed their medication management strategy during the observational period. The overall patient characteristics are described in Supplementary 1.

**Table 1:** Baseline characteristics and adherence metrics for eight patients prior to (pre) and during the COVID-19 lockdown, sorted by ascending taking adherence values (pre-lockdown)

ID	Baseline characteristics						Taking adherence [%]			Timing adherence [%]			Drug holidays	
	Age [y]	Sex	DOAC	MoCA	NIHSS	mRS	Pre	During	∆	Pre	During	∆	Pre	During
1	88	F	edoxaban	28	1	1	56.7	82.5	25.8	56.7	82.5	25.8	2	0
2	74	M	apixaban	26	2	2	71.1	69.8	− 1.3	71.1	67.4	− 3.6	0	1
3	86	F	dabigatran etexilat	25	1	2	86.6	82.1	− 4.6	82.1	79.5	− 2.7	1	0
4	75	M	apixaban	25	4	2	93.3	94.0	0.7	90.0	92.4	2.4	0	0
5	83	M	dabigatran etexilat	26	3	3	94.6	94.2	− 0.4	87.3	88.3	1.1	0	0
6	73	M	apixaban	28	2	2	97.1	96.1	− 1.1	94.6	96.1	1.4	0	0
7	80	M	rivaroxaban	28	0	2	99.1	100.0	0.9	99.1	98.6	− 0.5	0	0
8	84	F	apixaban	20	0	0	100.0	98.3	− 1.7	100.0	97.2	− 2.8	0	0

A negative difference (∆) indicates an adherence decline during compared to pre-lockdown

*DOAC* direct oral anticoagulation, *F* female, *M* male, *MoCA* Montreal Cognitive Assessment, *mRS* modified Rankin Scale, *NIHSS* National Institutes of Health Stroke Scale, *y* years, *∆* difference during—pre

### Adherence estimates and questions on medication intake behaviour

Patients had an average of 10.9 weeks (range 4.3–20.6) of adherence data prior to the lockdown, and 12.0 weeks (range 7.7–13.7) during the lockdown. The dot charts of electronic adherence data are presented in Fig. 1. From 1278 monitored days (608 days pre and 670 days during lockdown), we excluded 134 days (41 days [6.7%] pre and 93 days [13.9%] during lockdown) due to technical errors of the device (131 days) and physician-initiated DOAC pauses (3 days).

Three patients (ID 1–3) had pre-lockdown *taking* and *timing adherence* below 90% (mean 71.4% ± 15.0 and 70.0% ± 12.8), and two of them had at least one *drug holiday* pre-lockdown (Table 1). One patient (ID 1) showed a substantial increase in *taking* and *timing adherence* during the lockdown (both + 25.8%). This elderly female patient had two *drug holidays* pre-lockdown and no *drug holidays* during lockdown. She reported no major medical events nor changes in her life during the study that could explain the amelioration of her medication intake behaviour. For the remaining two patients (ID 2 and 3) with pre-lockdown *taking adherence* below 90%, a moderate decline in *taking* or *timing adherence* was observed during lockdown that corresponds to a difference (during—pre) greater than 3%. From the five patients with pre-lockdown *taking adherence* over 90% (mean 96.8% ± 2.9; ID 4–8), *taking* and *timing adherence* during lockdown remained stable below a difference value of 3%. None of these patients had *drug holidays*, neither pre- nor during lockdown.

All patients denied any change in their medication intake behaviour due to the imposition of the lockdown. Three patients spontaneously reported limitations in their daily life such as fewer visits from family members or death of friends due to COVID-19.

The sensitivity analysis including the electronic adherence data beyond June 19, 2020 of patients in whom the end of the lockdown occurred before the end of the observational phase (ID 2, 3, 4, 5, 8) yielded consistent findings with the main analysis (Supplementary 2).

## Discussion

To our knowledge, this is the first report on the impact of the COVID-19 lockdown on the adherence of DOAC-treated stroke patients. This secondary analysis of electronic adherence monitoring data from the ongoing MAAESTRO study provides a detailed insight in the medication intake behaviour prior to and during the COVID-19 lockdown in a small but well-characterized sample of stroke outpatients. The major finding of this analysis is that no disruptive effect from the imposition of lockdown measures was observed on the medication intake behaviour of patients with high pre-lockdown adherence. On the contrary, the lockdown seemed to have affected the medication intake behaviour of patients with suboptimal pre-lockdown adherence, either positively or negatively.

Overall, patient characteristics in this analysis are consistent with those published previously by Albert et al.[23] Most patients (*N* = 5) in our small sample showed high *taking* and *timing adherence* over 90%, which is similar to other studies using electronic adherence monitoring [17, 31, 32]. All patients with high pre-lockdown adherence maintained similarly high adherence rates during lockdown. This observation suggests that a robust daily routine with regular intakes, little time variation and no *drug holidays* might reflect strong medication habits embedded in fundamental daily routines such as mealtime, wake-up, and sleep routines[20], that are resistant to lockdown-induced changes.

On the contrary, in the minority of patients with a suboptimal adherence before the imposition of the lockdown (*Ν* = 3), we observed changes in medication intake behaviour during lockdown. Notwithstanding the limited number of patients, we observed both a decline in adherence metrics in two patients and—unexpectedly—a substantial increase in one. Although all patients reported that they were not aware of any relevant intentional changes in their medication intake behaviour triggered by or during lockdown, it seems that a major disruption of social life in the form of a lockdown might still affect adherence in patients with previously suboptimal adherence habits. Interestingly, it seems that this effect might be either negative (i.e., causing further disruption of medication habits) or positive (i.e., by potentially limiting other daily activities and thereby indirectly promoting a higher medication adherence). This, however, remains speculative and should be confirmed in larger, adequately powered studies.

So far, few studies have assessed the impact of the COVID-19 pandemic on medication adherence in patients with rheumatic and respiratory diseases. In these studies, adherence was measured with self-report and results were conflicting, with some studies reporting a negative effect, and some reporting no effect [33–37]. One study analysed controller inhaler use in asthma and chronic obstructive pulmonary disease patients whose devices were electronically tracked and who received an alert for missed doses [38]. A 14.5% increase in adherence to inhaler treatment was observed between January 2020 and March 2020. Considering the different adherence assessment methods, diseases and medications in those studies, no direct comparisons with our analysis are possible.

### Strengths

First, we used a standardized, previously established cleaning process of electronic adherence data, and excluded data gaps that were mostly due to technical failure. By doing this, we minimized errors and bias, and ensured constant data quality. Second, we opted for an intra-individual comparison of adherence data prior to and during the lockdown. Because medication intake behaviour and the factors that influence it are complex, our approach seems superior to a matched comparison of the adherence of different patients prior to and during lockdown, which might have included a larger patient sample but would have been subject to confounding through not quantifiable factors. Third, we performed a sensitivity analysis on an adjusted dataset with an extended monitoring period that yielded consistent results, pointing to the robustness of our findings. Fourth, we opted for at least four weeks of monitoring although more patients would have been eligible with a shorter observation time. However, by doing so, we increased the precision of our measures and thus, the power of our estimates. We claim that four weeks are sufficient to detect a deviation in behaviour.

### Limitations

We acknowledge some limitations. First, this was a secondary analysis from an ongoing study that was not pre-specified in the original study protocol, owing to the unforeseeable emergence of the COVID-19 pandemic. Second, the sample size is small, limiting us to a purely descriptive presentation of our findings. Thus, we urge to a cautious interpretation of our findings. Although 130 patients will be recruited for the MAAESTRO study over four years, only 12 patients were participating during the few months of the first pandemic wave in 2020 in Basel.

## Conclusion

Our observations based on electronic adherence monitoring in DOAC-treated stroke outpatients suggest that disruptions of social life such as the COVID-19 lockdown seem to have little effect on the medication intake behaviour of patients with a pre-established high adherence. On the contrary, in patients with suboptimal adherence more meaningful changes in their medication intake behaviour might occur.

## Supplementary Information

Below is the link to the electronic supplementary material.

Supplementary file1 (DOCX 19 KB)

## Data Availability

The data that support the findings of this study are available upon reasonable request from the corresponding author. The data are not publicly available due to privacy or ethical restrictions.
