# Supplementary material for: Impact of the COVID-19 lockdown on the adherence of stroke patients to direct oral anticoagulants: a secondary analysis from the MAAESTRO study
Source: J Neurol. 2021 Jun 3;269(1):19–25. doi: 10.1007/s00415-021-10631-5 (PMC8173508; doi:10.1007/s00415-021-10631-5)
Supplement: Supplementary file 1 — Supplementary file1 (DOCX 19 KB) [file 415_2021_10631_MOESM1_ESM.docx]

# Supplementary 1

**Table 1** - Baseline characteristics of the eight patients at study inclusion

| **Patient characteristics** |  |
| --- | --- |
| Age in years, median (IQR) | 81.5 (74.8 – 84.5) |
| Male, n (%) | 5 (62.5) |
| Employment status: retired, n (%) | 8 (100) |
| Education ≤12 years, n (%) | 7 (87.5) |
| Living alone, n (%) | 2 (25.0) |
| Comorbidities, n (%) |  |
| Hypertension | 7 (87.5) |
| Dyslipidemia | 7 (87.5) |
| Diabetes | 2 (25.0) |
| AF newly diagnosed during stroke hospitalization, n (%) | 2 (25.0) |
| CHA_2_DS_2_-VaSc score, mean ± SD | 5.6 ± 1.1 |
| eGFR in mL/min/1.73m^2^ mean ± SD | 60.3 ± 15.6 |
| eGFR <50mL/min/1.73m^2^, n (%) | 2 (25.0) |
| BMI in kg/m^2^, mean ± SD | 26.7 ± 5.8 |
| MoCA Score, median (IQR) | 26 (25 – 28) |
| Smoking status, n (%) |  |
| Current | 0 (0) |
| Previous | 4 (50.0) |
| **Stroke characteristics** | |
| Days of hospitalization, median (IQR) | 9.5 (6.5 – 10.0) |
| Days of inpatient rehabilitation, median (IQR) | 10.0 (0.0 – 21.8) |
| mRS, median (IQR) | 2.0 (1.8 – 2.0) |
| mRS ≤2, n (%) | 7 (87.5) |
| NIHSS, median (IQR) | 1.5 (0.8 – 2.3) |
| **Medication characteristics** |  |
| Total number of medications, median (IQR) | 6 (5 – 8) |
| OAC use prior to stroke, n (%) |  |
| DOAC | 1 (12.5) |
| VKA | 2 (25.0) |
| DOAC regimen at hospital discharge, n (%) |  |
| twice-daily | 6 (75.0) |
| once-daily | 2 (25.0) |
| DOAC agent at hospital discharge, n (%) |  |
| apixaban | 4 (50.0) |
| dabigatran etexilate | 2 (25.0) |
| rivaroxaban | 1 (12.5) |
| edoxaban | 1 (12.5) |
| Pillbox use during observational phase, n (%) | 5 (62.5) |
| Alarm use during observational phase, n (%) | 2 (25.0) |

Abbreviations: **AF:** atrial fibrillation, **BMI:** body mass index, **CHA_2_DS_2_-VaSc:** stroke risk score for patients with AF, **DOAC:** direct oral anticoagulation, **eGFR:** estimated glomerular filtration rate, **IQR:** interquartile range, **MoCA:** Montreal Cognitive Assessment, **mRS:** modified Rankin Scale, **NIHSS:** National Institutes of Health Stroke Scale, **OAC:** oral anticoagulation, **SD:** standard deviation, **VKA:** vitamin K antagonist

# Supplementary 2

**Table 2** **-** Adherence metrics for eight patients prior to (pre) and during the COVID-19 lockdown, sorted by ascending taking adherence values (pre-lockdown) – a sensitivity analysis over the entire observational phase, independently of the end of the lockdown. A negative difference (∆) indicates an adherence decline during compared to pre-lockdown.

| **ID** | **Taking adherence [%]** | | | **Timing adherence [%]** | | | **Drug holidays** | |
| --- | --- | --- | --- | --- | --- | --- | --- | --- |
|  | pre | during | ∆ | pre | during | ∆ | pre | during |
| 1 | 56.7 | 82.5 | 25.8 | 56.7 | 82.5 | 25.8 | 2 | 0 |
| 2 | 71.1 | 58.2 | -12.8 | 71.1 | 56.6 | -14.5 | 0 | 3 |
| 3 | 86.6 | 80.8 | -5.8 | 82.1 | 77.2 | -4.9 | 1 | 0 |
| 4 | 93.3 | 92.9 | -0.5 | 90.0 | 90.1 | 0.1 | 0 | 0 |
| 5 | 94.6 | 94.4 | -0.1 | 87.3 | 89.4 | 2.1 | 0 | 0 |
| 6 | 97.1 | 96.1 | -1.1 | 94.6 | 96.1 | 1.4 | 0 | 0 |
| 7 | 99.1 | 100.0 | 0.9 | 99.1 | 98.6 | -0.5 | 0 | 0 |
| 8 | 100.0 | 98.4 | -1.6 | 100.0 | 97.6 | -2.4 | 0 | 0 |

**∆:** difference during - pre
